# Cancer-associated fibroblasts as a potential novel liquid biopsy marker in cancer patients

**DOI:** 10.1186/s13046-025-03387-7

**Published:** 2025-04-21

**Authors:** Franziska Weber, Kim-Lea Reese, Klaus Pantel, Daniel J. Smit

**Affiliations:** 1 Institute of Tumor Biology, University Medical Center Hamburg-Eppendorf, Martinistraße 52, 20246 Hamburg, Germany; 2 European Liquid Biopsy Society (ELBS), Martinistraße 52, 20246 Hamburg, Germany

**Keywords:** Circulating cancer-associated fibroblasts, cCAFs, Liquid biopsy, Blood-based biomarkers

## Abstract

Cancer-associated fibroblasts (CAFs) are tissue residing cells within the tumor microenvironment (TME). Stromal CAFs have been shown to be associated with poor prognosis and tumor progression in several solid tumor entities. Although the molecular mechanisms are not fully understood yet, a critical role within the TME through direct interaction with the tumor cells as well as other cells has been proposed. While most studies on CAFs focus on stromal CAFs, recent reports highlight the possibility of detecting circulating CAFs (cCAFs) in the blood. In contrast to invasive tissue biopsies for stromal CAF characterization, liquid biopsy allows a minimally invasive isolation of cCAFs. Furthermore, liquid biopsy methods could enable continuous monitoring of cCAFs in cancer patients and therefore may present a novel biomarker for solid tumors. In this work, we present an overview of cCAF studies currently available and summarize the liquid biopsy techniques for cCAF isolation and detection. Moreover, the future research directions in the emerging field are highlighted and the potential applications of cCAFs as novel biomarkers for solid tumor patients discussed.

## Background

### Cancer-associated fibroblasts as a central component of the tumor microenvironment

Cancer-associated fibroblasts (CAFs) play an important role in the tumor microenvironment (TME). As a pivotal component of the stroma, fibroblasts are present in high numbers in the TME [1]. In addition to their physiological function as key players for extracellular matrix homeostasis and their involvement in wound healing, a strong link of dysregulated fibroblasts to multiple diseases including cancer has been established in the past [2].

Several studies have shown the tumor-promoting effect of CAFs [3]. CAFs can influence the TME through different mechanisms including direct interaction via cell-cell-adhesion, the promotion of immune escape mechanisms and the remodeling of the extracellular matrix [4, 5].

However, there are also reports on anti-tumorigenic CAFs that can act as negative regulators [6], warranting the requirement for further functional characterization of CAFs.

Recent studies have demonstrated that CAFs show a high proliferation rate and can induce the degradation and remodeling of the extracellular matrix, epithelial mesenchymal transition, angiogenic shift, metabolic reprogramming toward a reverse Warburg phenotype or promote stem cell trait achievement as compared with normal fibroblasts [7–10].

Moreover, CAFs play a role in the production and regulation of extracellular matrix components, such as collagen, hyaluronan and proteoglycans, supporting the supply of nutrients to the tumor through angiogenesis [11]. The influence of CAFs on tumor cells can be either directly (e.g. by cell-cell interaction) or indirectly mediated (e.g. by paracrine secretion of factors). CAFs influence, for example, CD8 + T cells, which are inhibited in their anti-tumor function by immune checkpoint ligand expression of CAFs [12]. CAFs can also exert indirect effects on cells within the tumor stroma through paracrine signaling as they secrete proteins such as chemokines (i.e. CXCL1), interleukins (e.g. IL-6) and growth factors (i.e. EGF) that influence immune response and proliferation [11].

Of note, inversely to the influencing factors described above, CAF differentiation and CAF functions can also be regulated by tumor cells, for example through paracrine secretion of tumor extracellular vesicles or other factors, highlighting the strong crosstalk within the TME [11]. The activation of CAFs can be influenced by cytokines (e.g. TGF-ß, IL-1, PDFG) that originate from cancer cells [11]. For example, the TGF-ß signaling pathway is particularly active in advanced pancreatic cancer and activated CAFs can also secrete TGF-ß [11, 13]. The activated CAFs produce pro-tumorigenic factors, which in turn promote cancer cell proliferation. In pancreatic ductal adenocarcinoma (PDAC), for example, cancer cells can release exosomes that support the recruitment and activation of CAFs. By secreting chemokines (e.g. CXCL-1) and interleukins (e.g. IL-6), CAFs contribute to an inhibitory immune microenvironment of the pancreas and promote pancreatic cancer tumor growth [11]. The interaction between tumor-associated macrophages and CAFs has also been shown in studies on prostate cancer [14]. CAFs promote the transformation to M2 macrophages and in this way enhance tumor progression [15].

### Molecular subtypes and heterogeneity of CAFs

Over the past years, a strong phenotypic and molecular heterogeneity of CAFs has been reported [16–18]. As a consequence, the functional role of CAFs includes both, tumor-promoting and tumor-inhibiting effects [19]. Initially, it has been assumed that CAFs originate from a homogenous population of stromal cells, but recent research suggests that several cell types in addition to fibroblasts can represent the origin of CAFs, including adipocytes, pericytes, endothelial cells, bone marrow-derived macrophages, among others [20, 21]. Therefore, to ensure precise identification of this heterogeneous population, it is paramount to use multiple markers including both CAF positive and CAF negative markers [22]. Common CAF positive markers are alpha smooth muscle actin (α-SMA), fibroblast activation protein (FAP), fibroblast specific protein (FSP) and vimentin, but many more markers have been examined and reviewed elsewhere [23]. Most studies on CAFs have been conducted in PDAC due to the strong desmoplastic stroma reaction which is one of the key histological characteristics of the tumor compared to other epithelial tumors [24]. Nevertheless, also in other solid tumor entities including lung cancer [25] and colorectal cancer [26], the presence and importance of tissue resident CAFs have been reported.

Cancer-associated fibroblasts can be divided into distinct molecular subtypes based on their diverse phenotype. The molecular subgroups can exert different functional impacts within the tumor microenvironment with either promoting, inhibiting or mixed effect on the tumor itself [4].

Various CAF subtypes have been reported based on the different expression of markers and their secretomes in solid tumor patients [27, 28], with myCAFs, iCAFs and apCAFs being the most commonly reported.

MyCAFs [29] are characterized by high expression of α-SMA and low expression of IL-6. Furthermore, they can exert myocontractile properties [29, 30]. In PDAC, it has been reported that myCAFs are close to the tumor site and are activated by direct contact with neoplastic cells [29, 31]. While most studies have implicated a tumor promoting role of myCAFs, some reports suggest that myCAFs can also contribute to tumor inhibition [4]. In contrast to myCAFs, iCAFs [29] are characterized by low α-SMA expression but high IL-6 secretion contributing to an inflammatory microenvironment. Moreover, it has been shown that iCAFs stimulate tumor cell proliferation via the secretion of inflammatory cytokines and growth factors [4, 32]. ApCAFs [33] are characterized by the expression of the Major Histocompatibility Complex II (MHC-II) which allows them to present antigens and interact with CD4 + T cells, thereby contributing to immunomodulation. While several reports underline the immunosuppressive effects and thus tumor promoting effect of apCAFs through regulatory T cell differentiation in pancreatic cancer and breast cancer [34, 35], T-cell immunity against the tumor has been reported in lung cancer [36], indicating a context and tumor-specific function of apCAFs [4]. In addition to these markers stated here, several other markers have been reported and reviewed elsewhere [37–40].

In addition to the diverse molecular subtypes, it has been shown that CAFs exhibit a dynamic and highly plastic phenotype that is interconvertible by differential activation of signaling pathways. One example is the regulation through the TGF-ß signaling pathway, in which iCAFs can become myCAFs [4] or apCAFs can change to myCAFs [33].

The highly heterogeneous origin, the high plasticity with the capacity to interchange subtypes and context as well as the tumor-dependent functional role represent challenges for studying CAFs in cancer patients [41].

### Circulating CAFs as novel liquid biopsy marker class

The sampling of body fluids, referred to as ‘liquid biopsy’ has opened and enabled novel strategies in cancer diagnostics and therapy [42]. Various body fluids, including blood, urine, saliva, cerebrospinal fluid and cyst fluid, can be used to detect circulating tumor cells (CTCs), other cancer-associated cells and tumor-derived products including circulating tumor DNA [43]. Particularly blood-based detection methods have received tremendous attention for detecting these biomarkers [43]. In addition, and in contrast to tissue biopsies, liquid biopsy is minimally invasive and repeatable, which allows monitoring the process throughout the disease [44]. The knowledge gained about the molecular tumor characteristics and its evolutions open new avenues for personalized cancer medicine [43].

While tissue resident CAFs have been thoroughly characterized over the last decade in several solid tumor entities, studies on the role of circulating CAFs (cCAFs) in the blood are emerging [45–52].

As liquid biopsy has several advantages over tissue biopsy [44], we have reviewed the currently available literature with respect to the isolation of cCAFs, the detection of cCAFs using different markers as well as the clinical applications of cCAFs and discuss the advantages and current challenges of cCAFs as novel liquid biopsy-based biomarkers in cancer patients.

## Methods

The literature search of studies investigating the role of cCAFs in the blood of cancer patients was performed in PubMed on 28th February 2025 with the following search strategy:

(“cCAF” OR cCAFs OR “circulating fibroblast*” OR “circulating cancer-associated fibroblast*” OR “circulating CAF*”) AND (“cancer” OR “tumor”) AND (“blood” OR “liquid biops*” OR “fluid biop*”) NOT (“fibronectin”) NOT “growth factor”.

The search resulted in 19 publications. After screening of abstracts, only original research studies for cCAF detection in the English language that used blood-based liquid biopsy methods were included in this review. 11 out of the 19 studies were excluded as they did not meet the inclusion criteria (not original research (*n* = 3), not blood (*n* = 2), not cCAFs (*n* = 5), not clinical research (*n* = 1)). The remaining 8 publications were eligible and therefore included in this review. Additional screening of the cited literature did not result in further candidates for inclusion. The following characteristics were extracted from the identified publications: tumor entity (breast cancer, prostate cancer, multi-cancer, local or metastatic), timepoint of blood draw, cohort details (cohort size, tumor stage), detection methods, biomarker details (detection markers, subtypes) and study details (author, year of publication, country).

### Circulating cancer-associated fibroblasts in the blood of cancer patients

**Fig. 1:**
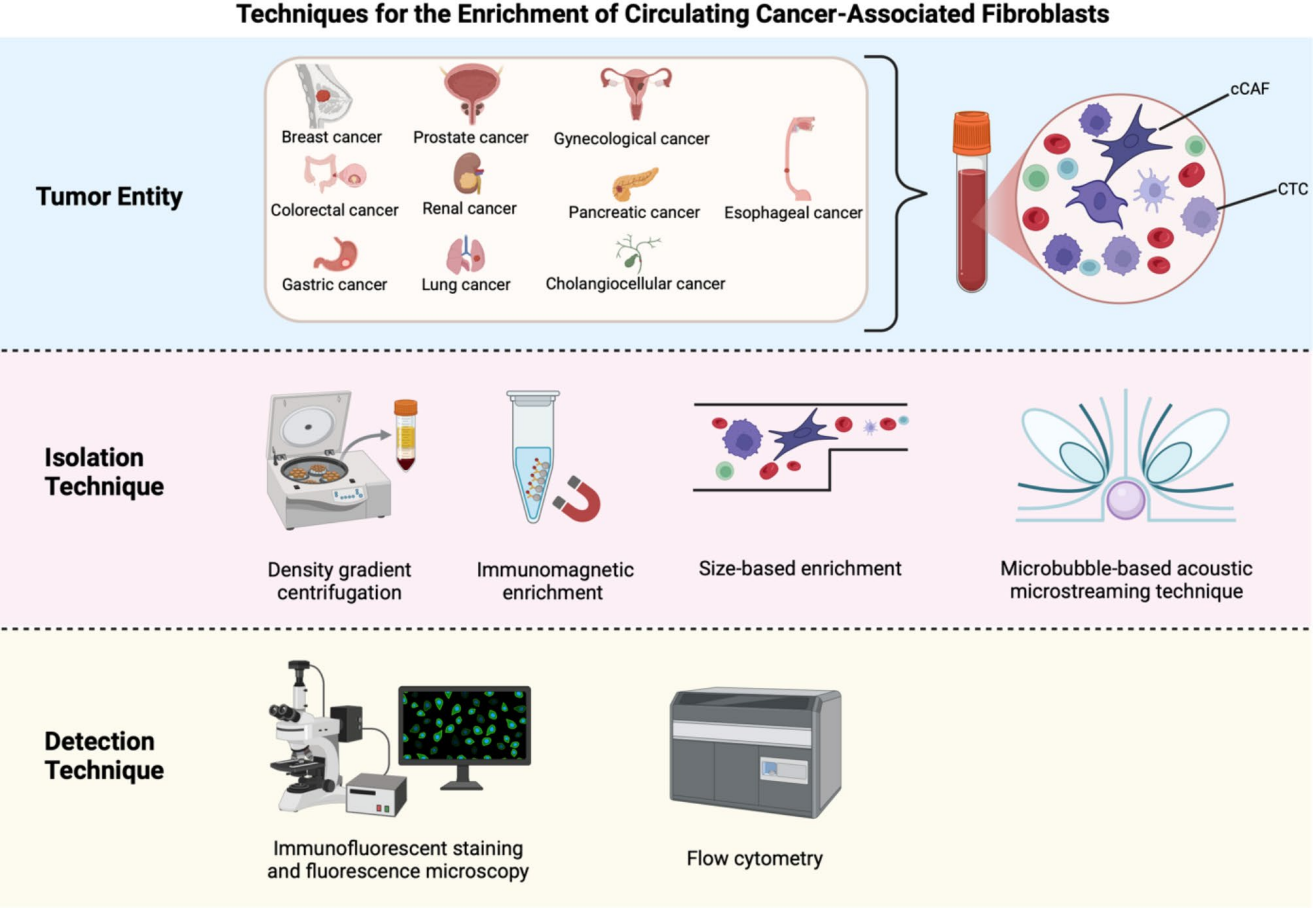
Overview of the cCAF enrichment and detection strategies used in the studies included in this review. Liquid biopsy methods enable the isolation of cCAFs (and other rare cells, e.g. CTCs) from patients’ blood samples. Various blood-based methods were used in the studies to isolate and detect the cells. The different approaches used different properties of the cCAFs which are depicted here (e.g. size, density, surface marker expression). Created in BioRender. Smit, D. (2025) https://BioRender.com/r17f728

Like other rare cells in the blood such as CTCs, the amount of cCAFs is low compared to the plethora of other blood cells [53, 54]. Therefore, their isolation is challenging and highly sensitive enrichment and detection methods are important [44]. cCAF isolation methods used today include density gradient centrifugation, immunomagnetic enrichment, size-based enrichment and microbubble-based acoustic microstreaming techniques, whereas cCAF detection is performed using fluorescence microscopy after immunofluorescent staining or flow cytometry **(**Fig. 1**)** [45–52].

In the following paragraphs, we present the identified studies through the literature search per cancer entity. An overview of the studies discussed can be found in Table 1.

**Table 1:** Overview of studies investigating the role of cCAFs in cancer patients

Tumor entity	Total *N* analyzed	Cohort	Time points of blood sample collection	Isolation technique	Detection technique	Identification markers	cCAF positivity rate (% of total)	Country	Publication year	Reference
**Breast cancer**	210	Non-metastatic: 184; metastatic: 26	Information not provided	Density gradient centrifugation	Immunofluorescent staining and imaging flow cytometry	DAPI+, α-SMA+, vimentin±, CK-, CD45-, CD31-	Non-metastatic: 4/184 (2.2%); metastatic: 3/26 (11.5%); overall: 7/210 (3.3%)	Poland	2023	[46]
**Breast cancer**	143	Stage I: 31; stage II/III: 52; stage IV: 60	Prior to or following primary treatment	Size-based microfilter technology	Immunofluorescent staining and fluorescence microscopy	DAPI+, FAP+, CK-, CD45-	Stage I: 23/31 (74%); stage II/III: 33/52 (63%); stage IV: 42/60 (70%); overall: 98/143 (68.5%)	USA	2021	[48]
**Breast cancer**	13	Non-metastatic: 5; metastatic: 8	Information not provided	Microbubble-based acoustic microstreaming technique	Immunofluorescent staining and fluorescence microscopy	DAPI+, FAP+, EpCAM-, CD45-	Non-metastatic: 2/5 (40%); metastatic: 5/8 (62.5%); overall: 7/13 (53.8%)	USA	2021	[49]
**Prostate cancer**	22	Non-metastatic: 10; metastatic: 12	Information not provided	Immunomagnetic enrichment by EpCAM	Immunofluorescent staining and fluorescence microscopy	DAPI+, vimentin+, CK8/18/19-, CD45-	Non-metastatic: 0/10 (0%); metastatic: 7/12 (58.3%); overall: 7/22 (31.8%)	USA	2012	[50]
**Prostate cancer**	18	Metastatic: 18	Before starting with the androgen deprivation therapy	Diagnostic leukapheresis	Immunofluorescent staining and flow cytometry	DAPI+, FAP+, EpCAM-, CD45±	Metastatic: 18/18 (100%)	Netherlands	2024	[51]
**Multi-tumor**	56	Breast cancer: non-metastatic: 13, metastatic 34; Prostate cancer: non-metastatic: 3; Colorectal cancer: metastatic: 6	Information not provided	Size-based microfilter technology	Immunofluorescent staining and fluorescence microscopy	DAPI+, FAP+, CK-, CD45-	Breast cancer: non-metastatic: 3/13 (23%), metastatic: 30/34 (88%); Prostate cancer: non-metastatic: 2/3 (66.7%); Colorectal cancer metastatic: 6/6 (100%)	USA	2015	[45]
**Multi-tumor**	45	Metastatic Colorectal cancer, Pancreatic cancer, Esophageal cancer, Gastric cancer, Prostate cancer, Breast cancer, Lung cancer, Gynecological cancers, Renal cancer	Longitudinal at different time points (pre-chemotherapy treatment, following 1 or 2 cycles of chemotherapy)	Immunomagnetic enrichment by anti-fibroblast microbeads	Immunofluorescent staining and fluorescence microscopy	DAPI+, α-SMA+, CD45-	Metastatic: 44/45 (98%)	USA	2020	[47]
**Multi-tumor**	45	Metastatic PDAC: 22; Metastatic gastrointestinal malignancies (mGI): 23 (colorectal carcinoma: 11, gastric cancer and gastroesophageal junction adenocarcinoma: 8, cholangiocellular carcinoma: 4)	Initial diagnosis or disease progression	Sequential assay combining marker-dependent enrichment with anti-fibroblast microbeads and size- and deformability-based enrichment	Immunofluorescent staining and fluorescence microscopy	DAPI+, α-SMA+ and/or FAP+, AE1/AE3-, CD45-	Metastatic PDAC: 21/22 (95.4%); mGI: 18/23 (78.2%)	Germany	2025	[52]

### Breast cancer

In total, three studies have assessed the role of cCAFs in the blood of breast cancer patients.

All three studies used CD45- and DAPI + as markers to identify cCAFs. Sharma et al. [48] and Jiang et al. [49]. both used FAP as an additional positive selection identification marker and Muchlińska et al. [46] used α-SMA. In addition, the studies by Muchlińska et al. [46] and Sharma et al. [48] both used CK and Jiang et al. [49] used EpCAM as negative markers to distinguish cCAFs from circulating epithelial cells/CTCs. All blood samples analyzed in the studies came from patients at different stages of breast cancer, including both early and advanced cases [46, 48, 49]. The numbers of CTCs and cCAFs were compared to healthy donors in two of the three studies [46, 49].

Jiang et al. [49] established a novel label-free method for simultaneous CTC, cCAF and immune cell isolation by acoustic microstreaming in a custom-made microfluidic device within just eight minutes while preserving the viability of the captured cells. Whole blood samples were subjected to red blood cell lysis and centrifuged, the pellet was resuspended and processed through the microfluidic device [49]. Cells that exceed a size of 16 μm were captured in the microfluidic devices and retrieved for subsequent immunofluorescent staining [49]. In spiking experiments using normal human lung fibroblasts in healthy donor blood, the authors could achieve a recovery rate of approximately 94% with a high viability rate of the trapped cells of 90% [49]. After technical validation using cell lines, the authors analyzed blood samples from 8 metastatic and 5 localized breast cancer patients as well as 5 healthy donors [49]. cCAFs were defined as DAPI+/FAP+/CD45- cells, whereas CTCs were defined as DAPI+/FAP-/CD45- cells [49]. The authors could detect cCAFs in 75% of localized cases and all metastatic breast cancer patients analyzed, whereas CTCs were only detected in around 71% of metastatic patients [49]. Interestingly, in two of the metastatic cases, heterotypic cCAF-CTC clusters could be detected, that were not present in locoregional advanced breast cancer patients [49]. In line with the data from other studies [51], the healthy donors analyzed did not have detectable cCAFs or CTCs [49]. In a next step, the authors used receiver operator curves (ROC) to evaluate the diagnostic accuracy of CTCs and cCAFs. Both, cCAFs and CTCs, showed a good diagnostic accuracy (AUC 0.889 and AUC 0.955, respectively) to discriminate healthy donors from cancer cases [49]. Of note, cCAFs outperformed CTCs with respect to the prediction of metastatic disease (AUC 0.975 versus AUC 0.675) in the breast cancer patients indicating the potential application of cCAFs as prognostic markers in breast cancer [49].

In a study by Muchlińska et al. [46], the authors recruited 210 breast cancer patients in different stages as well as healthy volunteers without cancer. A one-tube assay using peripheral blood utilizing immunofluorescent staining and imaging flow cytometry analysis of DAPI, α-SMA, CK, vimentin, CD29, CD31 and CD45 was used to simultaneously detect CTCs and cCAFs [46]. cCAFs were defined as α-SMA+/CK-/DAPI+/vimentin±/CD31-/CD45- or CD29+/CK-/DAPI+/vimentin±/CD31-/CD45- cells by the authors [46], although no cCAFs expressing CD29 were detected. In line with the data from Jiang et al. [49], all 20 healthy volunteers analyzed were negative for CTCs as well as cCAFs. Surprisingly, despite the high number of cCAF markers used for analysis and the extensive study cohort, only 7 out of 210 patients (3.33%) had detectable cCAFs in the blood, whereas CTCs were detected in 58 out of 210 patients (27.6%) using the one-tube assay [46]. Nevertheless, CTC as well as cCAF status were significantly associated with the presence of distant metastasis in breast cancer patients [46]. Moreover, the authors report that cCAFs coincided with CTCs and patients with higher CTC numbers were more likely to be positive for cCAFs [46]. With respect to the surprisingly low frequency of cCAF positive patients compared to other studies (e.g. Ao et al. [45]) the authors hypothesize that the low yield of cCAFs may be due to the high percentage of localized stages of breast cancer (as only 12% were metastatic cases) and on the other hand reported limitations with respect to the methodology that may have resulted in a loss of cells during the staining procedure [46].

In the study by Sharma et al. [48], blood samples from breast cancer patients across all stages (UICC stage I-IV) were used to detect cCAFs. Blood samples were processed through the FaCTChecker device, an automated size-based microfiltration enrichment system that was originally developed for CTC isolation [48]. Following the incubation, the filters were transferred to a glass slide for further immunocytochemistry staining. cCAFs were defined as DAPI+//FAP+/CK-/CD45- cells, whereas CTCs were defined as DAPI+/CK+/FAP-/CD45- cells [48]. The authors detected cCAFs in 74% of stage I (23 out of 31), 63% of stage II/III (*n* = 33 out of 52) and 70% of stage IV (*n* = 42 out of 60) pre-treated patients, whereas the absolute number of cCAFs was the highest in stage IV metastatic patients [48]. Interestingly, cCAF positivity rates exceeded CTC positivity rates ranging between 31% and 45% in the breast cancer cohort [48]. In addition to the pre-treated cohort, the authors also analyzed a cohort of treatment-naïve stage II and stage III breast cancer patients and warrant that cCAF and CTC counts may be underestimated in treated patients as CTCs were present in 88% and cCAFs in 76% of treatment-naïve breast cancer patients [48]. Moreover, the authors analyzed the presence of CTC and cCAF clusters. Homotypic cCAF-clusters consisting only of cCAFs were found more often than heterotypic cCAF-CTC clusters [48]. Strikingly, the authors could confirm the relevance of heterotypic cCAF-CTC clusters for metastasis in a murine orthotopic xenotransplantation model, with CD44 as an important mediator of cCAF-CTC interaction [48]. The study highlights the feasibility to detect cCAFs in breast cancer patients among all stages and furthermore defines the presence of heterotypic cCAF-CTC clusters as a poor prognosticator and potential metastasis promoting factor in breast cancer that should be evaluated in further studies [48].

### Prostate cancer

In two studies, cCAFs in the blood of prostate cancer patients were analyzed [50, 51]. Interestingly, these two publications are 12 years apart (2012 [50] vs. 2024 [51]). Besides the use of vimentin as a positive cCAF marker in both studies, different markers were used. While the study by Jones et al. [50] examined cCAFs in patients with localized and metastatic prostate cancer, the study by Booijink et al. [51] only included patients with metastatic disease.

Already in 2012, Jones et al. [50] published one of the first studies that reported cCAFs in blood samples. They analyzed blood samples from 12 metastatic prostate cancer patients, 10 patients with localized prostate cancer and 9 healthy individuals [50]. The blood samples were analyzed using the CellSearch system, which is an FDA-cleared system for CTC enumeration in epithelial tumors [55]. Here, the authors used the immunoaffinity based method to enrich cells by EpCAM expression and utilized the system for additional staining of CK, DAPI, CD45 and vimentin [50]. Cells that were neither positive for CK nor CD45, but positive for vimentin were considered as cCAFs [50]. Although EpCAM has not been described as a typical CAF marker, but rather as a marker for cells of epithelial origin, 7 out of 12 patients with metastatic prostate cancer had circulating fibroblast-like cells in their blood (enriched by EpCAM and DAPI+/CK-/CD45-/Vimentin + by immunostaining) [50]. Strikingly, patients with localized prostate cancer as well as healthy volunteers did not have detectable fibroblast-like cells in their blood. The number of cells in the blood of patients positive for these fibroblast-like cells ranged from 2 to 10 per 7.5 mL blood which is rather low compared to other studies [50]. Importantly, the presence of cCAFs correlated with known indicators of poor prognosis in prostate cancer including the presence of CTCs and high PSA levels [50]. However, the low cell counts can potentially be attributed to the use of EpCAM to enrich the cells for detection using the CellSearch system. To increase the sensitivity of the fibroblast-like cell detection, the authors suggest to use microfluidic devices or a directly targeted approach for fibroblasts [50], as other studies presented in this review have used [45, 48, 49].

In a second study on prostate cancer by Booijink et al. [51], blood samples from 18 metastatic prostate cancer patients were examined for the presence of rare cells including CTCs and cCAFs. If more than 2 CTCs per mL were detected in peripheral blood by CellSearch, diagnostic leukapheresis (DLA) was performed to obtain a sufficient yield of CTCs and cCAFs for further molecular characterization [51]. For cCAF detection cells derived from the DLA were stained with FAP, EpCAM and CD45, thereafter cells that were positive for FAP were collected by fluorescence activated cell sorting. The isolated cells were stained for collagen-I and vimentin and further functional assays for collagen-I secretion for molecular cCAF subtyping were conducted [51]. Analysis of the DLA products revealed cCAFs in all 18 samples from metastatic prostate patients, with the total number of FAP+/EpCAM- cCAFs ranging from 60 to 776 with a median of 360 cCAFs per 2 × 10^8^ mononuclear cells [51]. The CTC count ranged from 0 to 7436 with a median of 44 CTCs per 2 × 10^8^ mononuclear cells [51]. Surprisingly, FAP + cells were also found in 11 out of 12 healthy donor peripheral blood samples. Interestingly, this is the only study in which cCAFs were also detected in healthy study participants, although the number of detected cCAFs was lower (range: 0 to 71 per 2 × 10^8^ mononuclear cells) than in cancer patients [51]. The authors further divided identified cCAFs in FAP+/CD45 + and FAP+/CD45- subpopulations [51]. Morphological analysis revealed that FAP+/CD45 + cCAFs showed a spreading cytoplasm and a larger size compared to FAP+/CD45- cCAF. In cancer patients as well as healthy participants, more FAP+/CD45 + cCAFs were detected compared to FAP+/CD45- cCAFs [51]. The authors propose that the subpopulations could have different cellular origins, similar to the existing knowledge of tissue resident CAFs which other studies have already dealt with in the past [56, 57]. In the future, further studies should be carried out to differentiate between the two subpopulations and other cCAF subpopulation and their relevance in the clinical setting. With respect to the association of cCAFs and the clinical characteristics of the prostate cancer patients, no significant results were found which was mainly attributed to the limited sample size by the authors [51].

### Multi-tumor

In addition to the previously mentioned studies that have either focused their research exclusively on breast cancer [46, 48, 49] or prostate cancer patients [50, 51], three studies [45, 47, 52] have evaluated the role of cCAFs across several tumor entities including pancreatic cancer, colorectal cancer, renal cancer, lung cancer, gastric cancer and cholangiocellular cancer.

In the work of Ao et al. [45], the authors analyzed peripheral blood samples from local breast cancer, metastatic breast cancer, metastatic colorectal cancer, local prostate cancer patients and healthy donors. Blood samples were fixed with formalin, passed through their novel cell-size-based CTC isolation microfilter and immunofluorescent stained with pan-CK, CD45 and FAP [45]. In spike-in experiments with healthy donor blood and primary CAF cell lines, the authors reported a recovery rate of 95.0% with a low standard deviation of 2.8%.

For the analysis of patient samples, CTCs were classified as CK+/CD45- cells and cCAFs as FAP+/CK-/CD45- cells [45]. Moreover, in selected FAP + samples the authors demonstrated that α-SMA is also expressed confirming that the cells are indeed cCAFs [45]. All healthy volunteers analyzed were negative for CTCs as well as cCAFs similar to the data reported in other studies. The authors reported a significantly higher prevalence of cCAFs in metastatic breast cancer patients (88%) compared to localized breast cancer patients (23%) [45]. Similarly, the absolute number of cCAFs was significantly higher in the metastatic breast cancer group compared to those with local cancer [45]. Moreover, the authors could confirm their previous finding in colorectal cancer regarding the higher number of cCAF in the metastatic cases. Furthermore, the researchers observed CTC and cCAF clusters only in metastatic breast cancer patients and not in the samples of the non-metastatic patients or healthy volunteers [45]. Another interesting finding of the study is the fact that three metastatic cancer patients (two colorectal cancer patients and one breast cancer patient) did have detectable cCAFs, but no CTCs [45]. This finding underlines the importance and potential of additional cCAF detection to CTC detection as a potential biomarker for cancer prognosis. Moreover, given the limited knowledge, e.g. in colorectal cancer, the authors recommend that samples from other cancer entities should be analyzed [45].

A study by Ortiz-Otero et al. [47] analyzed blood samples from cancer patients with several metastatic cancers and healthy volunteers. For the first time, the presence of cCAFs in metastatic pancreatic, esophageal, gastric and renal cancer patients was shown [47]. The authors isolated mononuclear cells which were subsequently enriched by anti-fibroblast beads for cCAF isolation, however, the exact marker used in the commercially available kit is not publicly available [47]. CTCs were enriched by the depletion of CD45 + cells using anti-CD45 magnetic beads. Potential candidates were subjected to immunofluorescent staining for CTC and cCAFs. CTCs were defined as DAPI+/CK+/CD45- cells and cCAFs as DAPI+/α-SMA+/CD45- cells [47]. All healthy donors were negative for cCAFs. 44 out of 45 metastatic patients (98%) showed more than 17 cCAF per mL in their peripheral blood samples [47]. Among the samples of breast, prostate, colorectal and lung cancer patients, a higher number of cCAFs (more than 200 per mL) were found, whereas in patients with renal and gastric cancer, less than 60 cCAFs per mL blood were detected [47]. The study also investigated the use of cCAFs as a biomarker for therapy response in patients undergoing chemotherapy [47]. The numbers of cCAFs and CTCs were determined in 26 patients before, after the first and after the second chemotherapy cycle [47]. Although only 12% of the patients analyzed had a radiological progression of their disease, in this small subset of patients, the authors reported that consistently gradually increasing cCAF numbers could be detected. Moreover, cCAFs were found together with CTCs as a cluster. Ortiz-Otero et al. [47] postulate that the CTC-cCAF clusters may be an important precursor in cancer progression. This study also found that patients with cCAFs above the mean cCAF number had a significantly shorter overall survival compared to patients with low cCAFs, highlighting the potential and importance of cCAFs as prognosticators in patients with solid tumors undergoing chemotherapy [47].

In the work of Götze et al. [52], cCAFs were analyzed in patients with metastatic PDAC and patients with metastatic gastrointestinal malignancies, including gastric cancer, gastroesophageal junction adenocarcinoma, cholangiocellular carcinoma. After initial optimization assays comparing size- and deformability-based enrichment and marker-dependent enrichment techniques using primary human (tissue) CAF cell lines, the authors established a sequential liquid biopsy assay for cCAF isolation. The sequential assay combines the positive selection of cCAFs by magnetic cell separation with human anti-fibroblast microbeads (D7-FIB-conjugated) and the marker-independent size and deformability-based positive enrichment of the flow through from the cell separation. Thereafter, the cells were stained using antibodies against α-SMA, FAP, pan-keratin (AE1/AE3), CD105 and CD45 as well as with DAPI. In the study, cCAFs were defined as DAPI+, αSMA + and/or FAP+, AE1/AE3-, CD45- cells and CTCs were defined as DAPI+, αSMA-, FAPα-, AE1/AE3+, CD45- cells. CD105 was used as a marker to distinguish a recently reported subtype of CAFs with protumorigenic effects [52, 58]. In 95.4% of metastatic PDAC patients, cCAFs were detected in the blood samples, and in 78.2% of patients with metastatic gastrointestinal malignancies. The average number of cCAFs was significantly higher in PDAC patients (mean: 22.7, range: 0–72) compared to metastatic gastrointestinal malignancy patients (mean: 11.0, range: 0–65). In contrast, CTCs were detected in only 22.7% of metastatic PDAC patients and in only 30.4% of patients with gastrointestinal malignancies. Surprisingly, despite the metastatic stage, on average less than one CTC was detected in both patient groups. In addition, the authors compared the α-SMA and FAP expression in tissue with the number of detectable cCAFs in the blood, but could not demonstrate a correlation suggesting that the release may be independent of the tissue abundance [52].

An overview of the applied methods for cCAF isolation and their advantages and disadvantages is provided in Table 2.

**Table 2:** Overview of the different methodologies used for the cCAF enrichment, outlining their respective advantages and limitations

Method	Advantages	Disadvantages	Reference
Immunoaffinity-based enrichment	- Customizable - No large equipment required	- Markers need to be known - Cells with low expression may be lost - Heterogeneity of the desired cell population - Establishment and optimization required	[44, 59, 60]
Density gradient centrifugation	- Cost-effective method - Fast and standardized protocols - Suitable for large sample volumes	- No enrichment of the target cell population	[44, 59]
Size-based enrichment	- No specific marker required - Independent of phenotypes - Can enrich heterogenous populations - Fast protocol - Microfluidic platforms commercially available - High depletion of leucocytes and erythrocytes	- Other larger cells including CTCs are also enriched - Small-sized target cells may be lost	[44, 59, 61]
Microbubble-based acoustic enrichment	- No specific marker required - Very fast isolation - High viability of enriched cells	- Pre-processing of the sample by red blood cell lysis required - Custom-build - Not commercially available	[49]

### Synopsis: Tumor-agnostic and tumor-specific hallmarks of cCAFs as a novel liquid biopsy biomarker

cCAFs are a new emerging class of liquid biopsy markers. This section aims to provide a synopsis of the available knowledge derived from the clinical studies highlighting the similarities of cCAFs in different tumor entities and the tumor entity-specific characteristics which could leverage the discoveries in the field. Despite the wealth of information on CAFs in tumor tissues, only seven studies [45–52] have been published on the role of circulating CAFs in cancer patients (Table 1). The studies differ greatly in the number of participants, with the largest cohort comprising 230 patients [46] and the smallest only 18 patients [49] with the majority of data on cCAFs generated in cohorts of breast and prostate cancer. Nevertheless, cCAFs could also be found in other solid cancer entities including colorectal cancer, renal cell carcinoma and lung cancer [45, 47] although for some entities the sample size is limited and should be interpreted carefully. In the future, further research among other tumor entities should be conducted to acquire more knowledge on the role of cCAFs in different cancers.

The cCAF positivity rates in patients with cancer varied with a wide range between 3.3% [46] and 100% [51]. Although it has not been continuously reported, a strong trend of a higher positivity rate as well as higher absolute numbers of cCAFs are present in the metastatic setting which indicates that cCAF shedding may be tumor-stage dependent [45, 48, 49]. However, this statement is not universally applicable as observed in a cohort of breast cancer patients in which stage I had an even higher cCAF positivity rate than locally advanced cancer in stage II/III [48]. Detectable cCAFs at baseline have been shown to be associated with a poor prognosis and later stages in several cancers, and one study indicated that cCAFs may be used as suitable biomarkers for monitoring longitudinal treatment response in patients receiving chemotherapy [47]. In most studies [45–50], the studied healthy subjects did not have detectable cCAFs in the blood, suggesting a high specificity of cCAFs that may be also used as a diagnostic biomarker as demonstrated by ROC analysis with an AUC of 0.89 [49].

### cCAF heterogeneity and the selection of markers for enrichment

The combination of biomarkers for the identification of cCAFs varied among the studies. The most commonly used markers for the positive selection of cCAFs were FAP and α-SMA which were complemented in selected studies by other markers including vimentin, CD29 and CD31 [46, 50]. CK and CD45 were consistently used as exclusion markers to distinguish cCAFs, CTCs and remaining white blood cells [45, 48]. However, a high degree of heterogeneity of tissue resident CAFs has been reported in the past [11, 15]. Although several putative markers for cCAF detection and particular cCAF phenotyping including FAP, α-SMA and vimentin were assessed in the studies reported, the vast amount of potential markers and particularly secreted factors (e.g. IL-6) [4, 29] is not accounted for cCAF subtype identification at the moment. Moreover, as extensive molecular characterization of cCAFs isolated from blood is currently lacking, novel markers including ITGA-5, which was identified as a new biomarker for cCAF in a murine breast cancer xenograft model [62], could be utilized in the future to enhance cCAF capture rates and thereby increase prognostic value. With respect to the techniques used for cCAF isolation and detection, a wide spectrum has been utilized (e.g [49]. vs [51]). Although different techniques may have the advantage to detect the heterogeneous populations of cCAF, the lack of standardization makes it difficult to compare studies. This has been underlined by one of the studies in breast cancer that found surprisingly low cCAF numbers compared to the other studies [46]. In addition, the presence of cCAFs in the blood seems to be tumor-dependent as one of the multicancer studies found more cCAFs per mL blood in metastatic breast, lung, colorectal, prostate, esophageal and cervical cancer, than in gastric and renal cancer [47]. Therefore, cCAFs in different types of cancer should be examined in order to further understand the underlying factors that lead to cCAF shedding into the blood.

### cCAF clusters and the interaction of cCAFs with CTCs

In most of the studies, cCAFs were found as single cells, whereas four studies [45, 47–49] also detected cCAF clusters with other cCAFs or CTCs. Two studies have reported heterotypic cCAF-CTC clusters, which were only present in metastatic breast cancer patients, but not in localized cancer patients [45, 49]. Furthermore, Ortiz-Otero et al. [47] also found this type of cluster in metastatic stages of other tumor entities. In contrast, Sharma et al. [48] reported more homotypic cCAF-cCAF clusters rather than heterotypic cCAF-CTC clusters in breast cancer patient samples. Moreover, in a murine orthotopic xenotransplantation model, they demonstrated that CD44 plays an important role as a mediator for cCAF-CTC clusters [48]. Of note, these clusters could be associated with a poor prognosis, as they were only found in metastatic patients in the above-mentioned studies [45, 47–49]. Cluster detection could therefore also be an additional important indicator for cancer progression. It may also be interesting to note that most clusters were detected in breast cancer [48, 49] and none in prostate cancer [50, 51], which might indicate tumor-specific clustering. For CTCs, clustering with other cells in the blood has been reported in the past [63]. For example, for homotypic CTC clusters, Aceto et al. demonstrated that CTC clusters have an increased metastatic potential compared to single CTCs in a murine xenotransplantation model [64]. Nevertheless, also for other heterotypic clusters e.g. of CTCs with neutrophils [65] or platelets [66], tumor and metastasis promoting properties have been reported [63].

Overall, the studies indicate that the cCAF positivity rate and the absolute number of cCAF could be used as a very important indicator of the disease and its further prognosis. Compared to other liquid biopsy markers including ctDNA and extracellular vesicles, rare cells (e.g. CTCs and cCAFs) can be cultured and subsequently characterized regarding the molecular properties as well as their functional role [67]. For example, for CTCs, molecular characterization [67], ex vivo cultivation [68, 69], drug susceptibility testing [70, 71] and functional analysis [72, 73] have been conducted in the past that could provide valuable information for personalized therapy approaches. Of note, despite the lack of a clinical implementation, the prospective, randomized DETECT-III clinical trial has recently demonstrated the clinical benefit of lapatinib in addition to standard therapy after the molecular characterization of CTC in breast cancer patients with HER2- primary tumors but detectable HER2 + CTCs [74], highlighting the value of molecular characterization of rare cells in the blood.

### Outlook and future perspectives

Further understanding of the prognostic role, the functional role and the heterogeneity of cCAF could lead to the establishment of a novel viable analyte that can be sampled by minimally invasive liquid biopsy approaches. In the past, and exclusively for tissue resident CAFs, the potential of anti-CAF therapies, CAF depletion and CAF reprogramming have already been proposed [15]. Although the functional role of cCAFs is not clear yet, cCAFs may also represent a therapeutic target. To date, no clinical studies involving cCAFs for cancer diagnostics, risk stratification or therapeutic targeting are available in the ClinicalTrials database, however, more than 100 studies involving CAFs are registered. Knowledge from these studies could be leveraged for the contribution to the cCAF field. It is paramount to understand the functional role of cCAFs in the metastatic cascade and throughout the disease to elucidate whether cCAFs are a product of tumor growth and metastasis and just “bystanders” that can be detected in blood or if cCAFs are active contributors to the metastatic cascade due to crosstalk with other blood-borne cells as mediators with tumor-promoting functions. However, similar to the tissue resident CAFs, an extensive characterization of the molecular landscape of cCAFs is required in order to obtain the required knowledge for further therapeutic conclusions. cCAFs may emerge as valuable liquid biopsy analytes, as in contrast to other liquid biopsy analytes (i.e. extracellular vesicles, ctDNA, etc.) the cells are viable and could be used for functional assays. For example, similar to the establishment of stable CTC cell lines in the past decade [70, 75], in the future, cCAF cell lines could be established. The establishment of stable cell lines could then enable the analysis of the interaction with other cells and in-depth functional characterization [72]. More studies will follow with progressing technology and advances in the (tissue resident) CAF field that could contribute to the deeper understanding of cCAFs in the blood of cancer patients.

## Conclusion

Blood-based liquid biopsy enables simple and minimally invasive detection of cCAFs in contrast to tissue resident CAFs, providing a unique opportunity for early detection, risk stratification and longitudinal monitoring of cancer patients. Overall, the few available studies indicate that cCAFs are valuable prognosticators in cancer patients. In particular, an increased number of cCAFs was found in the blood of patients with metastatic cancer, but cCAF detection also succeeded in localized cases. Strikingly, it has been shown that cCAFs can be accompanied by other rare cells in the blood such as circulating tumor cells (CTCs), indicating a potential functional role that yet has to be elucidated. Studies that investigated cCAFs in addition to the CTC status suggest that for CTC-negative patients cCAFs analysis could improve risk stratification. For future studies, standardized protocols for cCAF isolation and additional highly sensitive markers for the detection of circulating CAFs would be useful. Importantly, as tumor-promoting and tumor-opposing effects have been reported, the molecular phenotype of cCAFs should be taken into account. Furthermore, as most studies at the moment have investigated breast and prostate cancer patients, cCAFs should be examined in other solid tumor entities in the future. In addition, the possibility of longitudinal monitoring of cCAFs during therapy should be evaluated to underline the role of cCAFs in cancer patients.

## Data Availability

No datasets were generated or analysed during the current study.

## Abbreviations

apCAF: Antigen-presenting CAF
AUC: Area under the curve
CAF: Cancer-associated fibroblast
cCAF: Circulating cancer-associated fibroblast
CD: Cluster of differentiation
CD29: Integrin beta-1
CK: Cytokeratin
CTC: Circulating tumor cell
CXCL-1: C-X-C motif chemokine ligand 1
DAPI: 4′,6-diamidino‐2‐phenylindole
DLA: Diagnostic leukapheresis
DNA: Deoxyribonucleic acid
ECM: Extracellular matrix
EGF: Epidermal growth factor
EpCAM: Epithelial cell adhesion molecule
FAP: Fibroblast activation protein
FSP: Fibroblast specific protein
HER2: Human epidermal growth factor receptor 2
iCAF: Inflammatory CAF
IL-6: Interleukin 6
ITGA-5: Integrin alpha 5
MHC-II: Major Histocompatibility Complex II
myCAF: Myofibroblast CAF
PDAC: Pancreatic ductal adenocarcinoma
PDGF: Platelet-derived growth factor
PSA: Prostate specific antigen
TGF-ß: Transforming growth factor beta
TME: Tumor microenvironment
VIM: Vimentin
α-SMA: Alpha smooth muscle actin

## References

[1] Quail DF, Joyce JA. Microenvironmental regulation of tumor progression and metastasis. Nat Med. 2013;19(11):1423–37. 24202395 10.1038/nm.3394 PMC3954707

[2] Plikus MV, et al. Fibroblasts: origins, definitions, and functions in health and disease. Cell. 2021;184(15):3852–72. 34297930 10.1016/j.cell.2021.06.024 PMC8566693

[3] Liu L, et al. Stromal myofibroblasts are associated with poor prognosis in solid cancers: A Meta-Analysis of published studies. PLoS ONE. 2016;11(7):e0159947. 27459365 10.1371/journal.pone.0159947 PMC4961396

[4] Yang D, et al. Cancer-associated fibroblasts: from basic science to anticancer therapy. Exp Mol Med. 2023;55(7):1322–32. 37394578 10.1038/s12276-023-01013-0 PMC10394065

[5] Yoshida GJ. Regulation of heterogeneous cancer-associated fibroblasts: the molecular pathology of activated signaling pathways. J Exp Clin Cancer Res. 2020;39(1):112. 32546182 10.1186/s13046-020-01611-0 PMC7296768

[6] Kalluri R. The biology and function of fibroblasts in cancer. Nat Rev Cancer. 2016;16(9):582–98. 27550820 10.1038/nrc.2016.73

[7] Eiro N et al. Gene expression profile of stromal factors in cancer-Associated fibroblasts from prostate cancer. Diagnostics (Basel), 2022. 12(7). 10.3390/diagnostics12071605 PMC9325062 35885510

[8] Eiro N et al. Analysis of the gene expression profile of stromal Pro-Tumor factors in Cancer-Associated fibroblasts from luminal breast carcinomas. Diagnostics (Basel), 2020. 10(11). 10.3390/diagnostics10110865 PMC7690699 33114046

[9] Eiro N et al. Breast cancer tumor stroma: cellular components, phenotypic heterogeneity, intercellular communication, prognostic implications and therapeutic opportunities. Cancers (Basel), 2019. 11(5). 10.3390/cancers11050664 PMC6562436 31086100

[10] Eiro N, et al. Cancer-associated fibroblasts affect breast cancer cell gene expression, invasion and angiogenesis. Cell Oncol (Dordr). 2018;41(4):369–78. 29497991 10.1007/s13402-018-0371-y PMC12995208

[11] Zhang T, et al. Cancer-associated fibroblasts in pancreatic ductal adenocarcinoma. Cell Death Dis. 2022;13(10):897. 36284087 10.1038/s41419-022-05351-1 PMC9596464

[12] Gorchs L, Kaipe H. Interactions between Cancer-Associated fibroblasts and T cells in the pancreatic tumor microenvironment and the role of chemokines. Cancers (Basel), 2021. 13(12). 10.3390/cancers13122995 PMC8232575 34203869

[13] Tjomsland V, et al. The desmoplastic stroma plays an essential role in the accumulation and modulation of infiltrated immune cells in pancreatic adenocarcinoma. Clin Dev Immunol. 2011;2011:212810. 22190968 10.1155/2011/212810 PMC3235447

[14] Comito G, et al. Cancer-associated fibroblasts and M2-polarized macrophages synergize during prostate carcinoma progression. Oncogene. 2014;33(19):2423–31. 23728338 10.1038/onc.2013.191

[15] Chen X, Song E. Turning foes to friends: targeting cancer-associated fibroblasts. Nat Rev Drug Discov. 2019;18(2):99–115. 30470818 10.1038/s41573-018-0004-1

[16] Özdemir BC, et al. Depletion of carcinoma-associated fibroblasts and fibrosis induces immunosuppression and accelerates pancreas cancer with reduced survival. Cancer Cell. 2014;25(6):719–34. 24856586 10.1016/j.ccr.2014.04.005 PMC4180632

[17] Hwang RF, et al. Cancer-associated stromal fibroblasts promote pancreatic tumor progression. Cancer Res. 2008;68(3):918–26. 18245495 10.1158/0008-5472.CAN-07-5714 PMC2519173

[18] Luo H, et al. Pan-cancer single-cell analysis reveals the heterogeneity and plasticity of cancer-associated fibroblasts in the tumor microenvironment. Nat Commun. 2022;13(1):6619. 36333338 10.1038/s41467-022-34395-2 PMC9636408

[19] Yamamoto Y, et al. The heterogeneity of cancer-associated fibroblast subpopulations: their origins, biomarkers, and roles in the tumor microenvironment. Cancer Sci. 2023;114(1):16–24. 36197901 10.1111/cas.15609 PMC9807521

[20] Sunami Y, Häußler J, Kleeff J. Cellular heterogeneity of pancreatic stellate cells, mesenchymal stem cells, and cancer-Associated fibroblasts in pancreatic cancer. Cancers (Basel), 2020. 12(12). 10.3390/cancers12123770 PMC7765115 33333727

[21] Huang X, et al. Oxidative stress induces monocyte-to-myofibroblast transdifferentiation through p38 in pancreatic ductal adenocarcinoma. Clin Transl Med. 2020;10(2):e41. 32508052 10.1002/ctm2.41 PMC7403727

[22] Kahounová Z, et al. The fibroblast surface markers FAP, anti-fibroblast, and FSP are expressed by cells of epithelial origin and May be altered during epithelial-to-mesenchymal transition. Cytometry A. 2018;93(9):941–51. 28383825 10.1002/cyto.a.23101

[23] Han C, Liu T, Yin R. Biomarkers for cancer-associated fibroblasts. Biomark Res. 2020;8(1):64. 33292666 10.1186/s40364-020-00245-w PMC7661188

[24] Masugi Y, The Desmoplastic Stroma of Pancreatic Cancer: Multilayered Levels of Heterogeneity, Clinical Significance, and, Opportunities T. Cancers (Basel), 2022. 14(13). 10.3390/cancers14133293 PMC9265767 35805064

[25] Lambrechts D, et al. Phenotype molding of stromal cells in the lung tumor microenvironment. Nat Med. 2018;24(8):1277–89. 29988129 10.1038/s41591-018-0096-5

[26] Li H, et al. Reference component analysis of single-cell transcriptomes elucidates cellular heterogeneity in human colorectal tumors. Nat Genet. 2017;49(5):708–18. 28319088 10.1038/ng.3818

[27] Bartoschek M, et al. Spatially and functionally distinct subclasses of breast cancer-associated fibroblasts revealed by single cell RNA sequencing. Nat Commun. 2018;9(1):5150. 30514914 10.1038/s41467-018-07582-3 PMC6279758

[28] Cords L, et al. Cancer-associated fibroblast classification in single-cell and Spatial proteomics data. Nat Commun. 2023;14(1):4294. 37463917 10.1038/s41467-023-39762-1 PMC10354071

[29] Öhlund D, et al. Distinct populations of inflammatory fibroblasts and myofibroblasts in pancreatic cancer. J Exp Med. 2017;214(3):579–96. 28232471 10.1084/jem.20162024 PMC5339682

[30] Geng X et al. Cancer-Associated fibroblast (CAF) heterogeneity and targeting therapy of CAFs in pancreatic cancer. Front Cell Dev Biology, 2021. 9. 10.3389/fcell.2021.655152 PMC8319605 34336821

[31] Chen Y, et al. Type I collagen deletion in αSMA(+) myofibroblasts augments immune suppression and accelerates progression of pancreatic cancer. Cancer Cell. 2021;39(4):548–e5656. 33667385 10.1016/j.ccell.2021.02.007 PMC8423173

[32] Bhattacharjee S et al. Tumor restriction by type I collagen opposes tumor-promoting effects of cancer-associated fibroblasts. J Clin Invest, 2021. 131(11). 10.1172/JCI146987 PMC8159701 33905375

[33] Elyada E, et al. Cross-Species Single-Cell analysis of pancreatic ductal adenocarcinoma reveals Antigen-Presenting Cancer-Associated fibroblasts. Cancer Discov. 2019;9(8):1102–23. 31197017 10.1158/2159-8290.CD-19-0094 PMC6727976

[34] Costa A, et al. Fibroblast heterogeneity and immunosuppressive environment in human breast cancer. Cancer Cell. 2018;33(3):463–e47910. 29455927 10.1016/j.ccell.2018.01.011

[35] Huang H, et al. Mesothelial cell-derived antigen-presenting cancer-associated fibroblasts induce expansion of regulatory T cells in pancreatic cancer. Cancer Cell. 2022;40(6):656–73.e7. 35523176 10.1016/j.ccell.2022.04.011 PMC9197998

[36] Kerdidani D et al. Lung tumor MHCII immunity depends on in situ antigen presentation by fibroblasts. J Exp Med, 2022. 219(2). 10.1084/jem.20210815 PMC8764966 35029648

[37] Pompella L, et al. Pancreatic cancer molecular classifications: from bulk genomics to single cell analysis. Int J Mol Sci. 2020;21(8):2814. 32316602 10.3390/ijms21082814 PMC7215357

[38] Malchiodi ZX, Weiner LM. Understanding and targeting natural killer Cell-Cancer-Associated fibroblast interactions in pancreatic ductal adenocarcinoma. Cancers. 2021;13(3):405. 33499238 10.3390/cancers13030405 PMC7865209

[39] Mhaidly R, Mechta-Grigoriou F. Fibroblast heterogeneity in tumor micro-environment: role in immunosuppression and new therapies. Semin Immunol. 2020;48:101417. 33077325 10.1016/j.smim.2020.101417

[40] Brichkina A, et al. A quick guide to CAF subtypes in pancreatic cancer. Cancers. 2023;15(9):2614. 37174079 10.3390/cancers15092614 PMC10177377

[41] Chen Y, McAndrews KM, Kalluri R. Clinical and therapeutic relevance of cancer-associated fibroblasts. Nat Rev Clin Oncol. 2021;18(12):792–804. 34489603 10.1038/s41571-021-00546-5 PMC8791784

[42] Pantel K, Alix-Panabieres C. Circulating tumour cells in cancer patients: challenges and perspectives. Trends Mol Med. 2010;16(9):398–406. 20667783 10.1016/j.molmed.2010.07.001

[43] Alix-Panabières C, Pantel K. Liquid biopsy: from discovery to clinical application. Cancer Discov. 2021;11(4):858–73. 33811121 10.1158/2159-8290.CD-20-1311

[44] Smit DJ, Pantel K. Circulating tumor cells as liquid biopsy markers in cancer patients. Mol Aspects Med. 2024;96:101258. 38387225 10.1016/j.mam.2024.101258

[45] Ao Z, et al. Identification of cancer-Associated fibroblasts in Circulating blood from patients with metastatic breast cancer. Cancer Res. 2015;75(22):4681–7. 26471358 10.1158/0008-5472.CAN-15-1633

[46] Muchlińska A et al. Improved characterization of Circulating tumor cells and cancer-Associated fibroblasts in One-Tube assay in breast cancer patients using imaging flow cytometry. Cancers (Basel), 2023. 15(16). 10.3390/cancers15164169 PMC10453498 37627197

[47] Ortiz-Otero N, et al. Chemotherapy-induced release of circulating-tumor cells into the bloodstream in collective migration units with cancer-associated fibroblasts in metastatic cancer patients. BMC Cancer. 2020;20(1):873. 32917154 10.1186/s12885-020-07376-1 PMC7488506

[48] Sharma U, et al. Heterotypic clustering of Circulating tumor cells and Circulating cancer-associated fibroblasts facilitates breast cancer metastasis. Breast Cancer Res Treat. 2021;189(1):63–80. 34216317 10.1007/s10549-021-06299-0

[49] Jiang R, et al. Rapid isolation of Circulating cancer associated fibroblasts by acoustic microstreaming for assessing metastatic propensity of breast cancer patients. Lab Chip. 2021;21(5):875–87. 33351008 10.1039/d0lc00969e

[50] Jones ML, et al. Circulating fibroblast-like cells in men with metastatic prostate cancer. Prostate. 2012;73(2):176–81. 22718300 10.1002/pros.22553 PMC3482413

[51] Booijink R et al. Identification of functional and diverse Circulating cancer-associated fibroblasts in metastatic castration‐naïve prostate cancer patients. Mol Oncol, 2024. 10.1002/1878-0261.13653 PMC12234390 38634185

[52] Götze J, et al. Identification and characterization of tumor and stromal derived liquid biopsy analytes in pancreatic ductal adenocarcinoma. J Exp Clin Cancer Res. 2025;44(1):14. 39815324 10.1186/s13046-024-03262-x PMC11737273

[53] Barradas AM, Terstappen LW. Towards the biological Understanding of CTC: capture technologies, definitions and potential to create metastasis. Cancers (Basel). 2013;5(4):1619–42. 24305653 10.3390/cancers5041619 PMC3875957

[54] Smit DJ, Schneegans S, Pantel K. Clinical applications of Circulating tumor cells in patients with solid tumors. Clin Exp Metastasis, 2024. 10.1007/s10585-024-10267-5 PMC11374849 38281256

[55] Ma L, et al. Liquid biopsy in cancer current: status, challenges and future prospects. Signal Transduct Target Ther. 2024;9(1):336. 39617822 10.1038/s41392-024-02021-w PMC11609310

[56] Ricci B et al. Osterix-Cre marks distinct subsets of CD45- and CD45 + stromal populations in extra-skeletal tumors with pro-tumorigenic characteristics. Elife, 2020. 9. 10.7554/eLife.54659 PMC7428306 32755539

[57] Kraman M, et al. Suppression of antitumor immunity by stromal cells expressing fibroblast activation protein-alpha. Science. 2010;330(6005):827–30. 21051638 10.1126/science.1195300

[58] Hutton C, et al. Single-cell analysis defines a pancreatic fibroblast lineage that supports anti-tumor immunity. Cancer Cell. 2021;39(9):1227–e124420. 34297917 10.1016/j.ccell.2021.06.017 PMC8443274

[59] Soda N, et al. Advanced liquid biopsy technologies for Circulating biomarker detection. J Mater Chem B. 2019;7(43):6670–704. 31646316 10.1039/c9tb01490j

[60] Nicolazzo C, et al. EpCAM(low) Circulating tumor cells: gold in the waste. Dis Markers. 2019;2019:p1718920. 10.1155/2019/1718920 PMC6766153 31636732

[61] Miller MC, et al. The parsortix™ cell separation System-A versatile liquid biopsy platform. Cytometry A. 2018;93(12):1234–9. 30107082 10.1002/cyto.a.23571 PMC6586069

[62] Lu T et al. In vivo detection of Circulating Cancer-Associated fibroblasts in breast tumor mouse xenograft: impact of tumor stroma and chemotherapy. Cancers, 2023. 15(4). 10.3390/cancers15041127 PMC9954095 36831470

[63] Aceto N. Bring along your friends: homotypic and heterotypic Circulating tumor cell clustering to accelerate metastasis. Biomedical J. 2020;43(1):18–23. 32200952 10.1016/j.bj.2019.11.002 PMC7090281

[64] Aceto N, et al. Circulating tumor cell clusters are oligoclonal precursors of breast cancer metastasis. Cell. 2014;158(5):1110–22. 25171411 10.1016/j.cell.2014.07.013 PMC4149753

[65] Szczerba BM, et al. Neutrophils escort Circulating tumour cells to enable cell cycle progression. Nature. 2019;566(7745):553–7. 30728496 10.1038/s41586-019-0915-y

[66] Labelle M, Begum S, Hynes RO. Direct signaling between platelets and cancer cells induces an Epithelial-Mesenchymal-Like transition and promotes metastasis. Cancer Cell. 2011;20(5):576–90. 22094253 10.1016/j.ccr.2011.09.009 PMC3487108

[67] Alix-Panabieres C, Pantel K. Circulating tumor cells: liquid biopsy of cancer. Clin Chem. 2013;59(1):110–8. 23014601 10.1373/clinchem.2012.194258

[68] Zhang L, et al. The identification and characterization of breast cancer CTCs competent for brain metastasis. Sci Transl Med. 2013;5(180):180ra48. 23576814 10.1126/scitranslmed.3005109 PMC3863909

[69] Cayrefourcq L, et al. Establishment and characterization of a cell line from human Circulating colon cancer cells. Cancer Res. 2015;75(5):892–901. 25592149 10.1158/0008-5472.CAN-14-2613

[70] Smit DJ, Pantel K, Jücker M. Circulating tumor cells as a promising target for individualized drug susceptibility tests in cancer therapy. Biochem Pharmacol. 2021;188:114589. 33932470 10.1016/j.bcp.2021.114589

[71] Yu M, et al. Cancer therapy. Ex vivo culture of Circulating breast tumor cells for individualized testing of drug susceptibility. Science. 2014;345(6193):216–20. 25013076 10.1126/science.1253533 PMC4358808

[72] Eslami-S Z, et al. Functional analysis of Circulating tumour cells: the KEY to understand the biology of the metastatic cascade. Br J Cancer. 2022;127(5):800–10. 35484215 10.1038/s41416-022-01819-1 PMC9427839

[73] Smit DJ, et al. Analysis of the plasticity of Circulating tumor cells reveals differentially regulated kinases during the Suspension-to-Adherent transition. Cancer Med. 2024;13(20):e70339. 39425449 10.1002/cam4.70339 PMC11489281

[74] Fehm T, et al. Efficacy of lapatinib in patients with HER2-Negative metastatic breast cancer and HER2-Positive Circulating tumor Cells—The DETECT III clinical trial. Clin Chem. 2024;70(1):307–18. 38175595 10.1093/clinchem/hvad144

[75] Shimada Y et al. Cell lines of Circulating tumor cells: what is known and what needs to be resolved. J Pers Med, 2022. 12(5). 10.3390/jpm12050666 PMC9148030 35629089

